# Atypical ubiquitin ligase RNF31: the nuclear factor modulator in breast cancer progression

**DOI:** 10.1186/s12885-016-2575-8

**Published:** 2016-07-26

**Authors:** Jian Zhu, Ting Zhuang, Huijie Yang, Xin Li, Huandi Liu, Hui Wang

**Affiliations:** 1 Research Center for Immunology, School of Laboratory Medicine, Henan Collaborative Innovation Center of Molecular Diagnosis and Laboratory Medicine, Xinxiang Medical University, Xinxiang, 453003 Henan Province People’s Republic of China; 2 Department of Biochemistry, University of Texas Southwestern Medical Center, Dallas, TX 75390 USA

**Keywords:** RNF31, Breast cancer, Ubiquitin ligase, Estrogen

## Abstract

Breast cancer causes the No.1 women cancer prevalence and the No.2 women cancer mortality worldwide. Nuclear receptor/transcriptional factor signaling is aberrant and plays important roles in breast cancer pathogenesis and evolution, such as estrogen receptor α (ERα/ESR1), tumor protein p53 (p53/TP53) and Nuclear factor kappa B (NFκB). About 60–70 % of breast tumors are ERα positive, while approximate 70 % of breast tumors are P53 wild type. Recent studies indicate that nuclear receptors/transcriptional factors could be tightly controlled through protein post-translational modification.

The nuclear receptors/transcriptional factors could endure several types of modifications, including phosphorylation, acetylation and ubiquitination. Compared with the other two types of modifications, ubiquitination was mostly linked to protein degradation process, while few researches focused on the functional changes of the target proteins. Until recent years, ubiquitination process is no longer regarded as merely a protein degradation process, but aslo treated as one kind of modification signal.

As an atypical E3 ubiquitin ligase, RNF31 was previously found to facilitate NFκB signaling transduction through linear ubiquitination on IKK_γ_(IκB kinase γ). Our previous studies showed important regulatory functions of RNF31 in controlling important oncogenic pathways in breast cancer, such as ERα and p53. This review highlights recent discoveries on RNF31 functions in nuclear factor modifications, breast cancer progression and possible therapeutic inhibitors targeting RNF31.

## Background

Breast cancer is one of the most frequent neoplastic lethality among women [1]. According to the receptor status classification based on estrogen receptor (ER), progesterone receptor (PR) and human epidermal growth factor receptor 2 (HER-2) positivity, breast cancer can be divided into luminal A, luminal B, HER2 type and triple negative/basal - like subtype [2]. Among these subtypes, luminal A and B could be treated with ERα antagonists and/or aromatase inhibitors, while HER2 enriched subtypes could be effectively controlled through Trastuzumab, a specific antibody for HER2 [3, 4]. Due to a lack of validated drug targets for triple negative/basal-like subtype, chemotherapy is the primary treatment for this group with the worst prognosis [5].

According to the oncogene addiction theory, each subtype of breast cancer needs at least one oncogenic pathway to maintain its survival. For the luminal A and B subtypes, estrogen signaling has the function to maintain breast cancer survival and malignant phenotype, while HER2 subtype is dependent on HER2 amplification/overexpression [6, 7]. As to the triple negative/basal-like subtype, the addictive oncogenic pathway is not totally clear. However, there were reports that NFκB and EGFR signaling were necessary to facilitate breast cancer progression [8–12].

Currently, few nuclear receptors/transcriptional factors lead to successful drug development and clinical applications. Since the post-translational modification on nuclear receptor/transcriptional factors was proved to be the key mechanism in regulating the relative intensity of cellular signaling [13], more studies start to focus on the exploration of biological functions on the nuclear factors modulators [14]. The increased knowledge of nuclear factor modulators will lay a solid foundation for selective targets on these modulation proteins and subsequently clinical applications.

The nuclear receptors/transcriptional factors could be subjected to several post-translational modifications, such as acetylation, methylation, phosphorylation and ubiquitination. Compared with other modifications, ubiquitination is processed sequentially via multiple ubiquitin ligases E1, E2 and E3, which was first recognized as the signal for protein destruction [15]. But further studies revealed that ubiquitination linked to signaling transduction and proper protein functioning [16]. A lot of non-destructive ubiquitination is ligated by the E3 ubiqutin ligases belonging to ring finger protein (RNF) family [17]. As one of the RNF family member, RNF31 (other names: HOIP; ZIBRA) was first cloned from breast cancer cell line and was identified as a classical component in linear ubiquitin assembly complex (LUBAC) to facilitate NFκB signaling transduction [18]. Our previous studies identified the oncogenic role of RNF31 in facilitating estrogen signaling and suppressing P53 pathway in breast cancers [19, 20]. Here we want to review the current knowledge about RNF31 as an ubiquitin ligase in breast cancer cell progression.

### E3 ubiquitin ligase and cancer

E3 ubiquitin ligases function to catalyze the transfer of ubiquitin from an E2 ubiquitin-conjugating enzyme to the lysine of a protein substrate. Ubiquitin molecules are attached to lysine residues on substrates via lysine residues on ubiquitin [21]. Different forms of ubiquitination have been identified such as mono-ubiquitination and poly-ubiquitination [15]. Mono-ubiquitination can be viewed as a necessary process for poly-ubiquitination or a separate event [22, 23]. Mono-ubiquitination is demonstrated to link to a change of substrate functions such as signal transduction or protein trafficking in addition to protein degradation [23]. For example, mono-ubiquitination of histone 2A (H2AX) by RNF8 is a necessary step of the DNA repair response [24]. Poly-ubiquitination has different lysine residues on ubiquitin protein as points of ubiquitination, including K63, K48, K27, K29, K33, K11 and linear ubiquitination [25–27]. The K48 and K63 ubiquitination process is related to proteasome dependent degradation [15]. However, the other atypical forms of ubiquitin, such as K27, K11 and linear ubiquitin, are less well understood, while there are accumulating evidences showing that they are involved in DNA repair, signal transduction and protein trafficking [15, 28, 29].

Beside to the classification of lysine ubiquitination sites, E3 ubiquitin ligases can also be divided by their functional domains, which include the HECT (homologous to the E6-AP carboxyl terminus) group and the RING finger group [21]. There are about 30 different HECT E3 ligases in mammals that are involved in protein transfer, immune reaction, and DNA damage response [21]. In general, the HECT family of E3 ligases is composed of two functional domains. The functional domain at the C-terminus is responsible for the interaction with E2 and ubiquitin molecules, while the N-terminal domain is responsible for substrate interaction [21]. One group of proteins, which belong to the HECT family are the SMURF (Smad ubiquitinylation regulatory factor) proteins, which regulate TGFβ and bone morphogenetic protein (BMP) signaling [30]. SMURF proteins interact with Smad proteins and regulate its poly-ubiquitination and degradation via the HECT domain. This process negatively controls the protein levels of the Smad proteins and subsequently controls TGFβ pathway output. There are about 700 different RING E3 ligases, most of which are not well studied [31]. According to the current knowledge, the functions of RING E3 ligases cover multiple aspects of cell physiological functions, including cell proliferation, cell migration, DNA damage, and protein trafficking [29, 31, 32]. Many of the RING E3 ligases are found to be involved in carcinogenesis [33]. BRCA1 is the most thoroughly studied RING E3 ligase in cancer. As a tumor suppressor protein, BRCA1 is shown to regulate gene expression, DNA repair after double stain break and protein ubiquitination [34]. ERα has been suggested as a putative BRCA1 target and BRCA1 inhibits ERα function [35]. Defects in BRCA1 ligase functions will lead to loss of the DNA repair response [36]. BRCA1 mutations are found in about 70 % of familial breast cancer and ovarian cancer [37]. In addition, recent studies showed that RNF54 (RBCK1) interacts with ERα and facilitates ERα target genes transcription [38]. Analysis of publically available data sets indicates that RBCK1 expression correlates with poor tamoxifen response [39].

### RNF31 as an E3 ubiquitin ligase

**Fig. 1:**
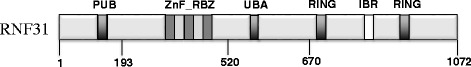
RNF31 protein domain structure. PUB domain, putative ubiquitin binding domain; ZNF-RBZ domain, Zinc finger domain in Ran-binding proteins and other proteins; UBA domain, ubiquitin binding associated domain; RING-IBR-RING domain, ring finger domain-in between RING-ring finger domain

Ring finger protein 31 (RNF31), also named HOIL-1-interacting protein (HOIP), was first cloned in 2004 from MCF-7 cells [40]. Figure 1 shows the domain structure of the RNF31 protein [41]. The PUB domain (putative ubiquitin binding domain) at the N-terminal is reported to bind cofactors [42]. The ZNF-RBZ domain (Zinc finger domain in Ran-binding proteins and other proteins) is related to the ubiquitin binding function [43]. The UBA domain (ubiquitin binding associated domain) has been shown to bind RBCK1 and mediate linear ubiquitination of IKKγ, which facilitates signal transduction of NFκB [44]. The RING-IBR-RING domain (RBR domain) at the C-terminal is thought to be the most important one for its ubiquitin ligase function [45]. The deletion of this domain will lead to loss of function of its substrates, such as IKKγ [41, 46].

**Fig. 2:**
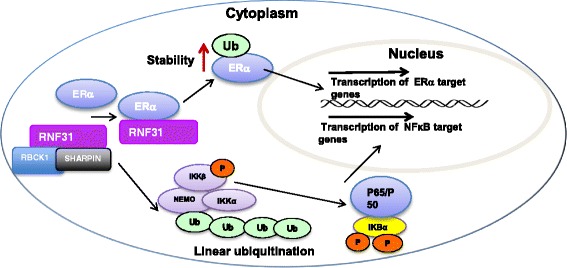
The proposed model for RNF31 effect on ERα signaling in breast cancer. RNF31 interacts with ERα and increases its stability possibility through the mono-ubiquitination manner. The stabilized ERα protein will enhance the estrogen dependent signaling transduction

RNF31 is highly expressed in muscle, heart, and testis [41]. In cells, RNF31 mainly localizes to the cytoplasm. Whole-body knockout of RNF31 will lead to embryonic lethality through TNFR1-mediated endothelial cell death [47]. The most well studied function of RNF31 is that it together with RBCK1 and SHARPIN, forms the linear ubiquitin chain assembly complex (LUBAC) which facilitates linear ubiquitination of IKKγ and NFκB signaling transduction as demonstrated in several conditional knockout mice models [46, 47] (Fig. 2). For example, conditional deletion of the RBR domain in B cells (B-HOIP^Δlinear^) leads to lack of or reduced NFκB and ERK signaling. Phenotypically, lack of RBR domain of RNF31 causes development deficiency of B cells and deficient thymus-dependent and thymus-independent antigen response [47]. RNF31 is also observed to contribute to inborn human immunity disorders, in which RNF31 missense mutation at PUB domain gives rise to the de-stabilized LUBAC complex and subsequently causes the auto-inflammation and immunodeficiency [48]. In addition, RNF31 is reported to modify ERK and JNK pathways leading to cisplatin resistance [49].

### RNF31 in human cancer

Although RNF31 was firstly cloned from MCF-7 cells, it was not instantly studied in cancer area. The most important reason is that the functional domain as well as its molecular function of RNF31 is not clear. As RNF31 was characterized as an E3 ubiquitin ligase in NFκB signaling transduction from these immunological and biochemical studies, more and more researchers started to focus on RNF31 function in cancer area. Firstly RNF31 is highly expressed in several human cancers, while RNF31 gene harbors low mutation frequency from the TCGA database (http://cbioportal.org/) [18]. Although RNF31 mutation/nucleotide variation is rare, RNF31 single-nucleotide polymorphisms were reported to highly activate LUBAC activity and contribute to occurrence of large B-cell lymphoma [50]. In the mechanistic studies, RNF31 was shown to interact and trans-repress DAX function, which facilitated carcinogenesis of adrenocortical carcinoma [51]. Besides RNF31 was also involved in chemotherapy outcome, Mackay et al reported that RNF31 contributed to cisplatin resistance through NFκB and JNK pathway [52]. Based on the current studies, RNF31 seems to act as an oncogene, while it may exert its function through two different models. One is that RNF31 contributes its carcinogenic role by facilitating NFκB pathway, which is already shown to be the key oncogenic pathway in several cancers. Another is that RNF31 might act as an oncogene through modulating these key nuclear receptors/transcription factors, such as ERα and P53.

### RNF31 in ERα and P53 signaling in breast cancers

**Fig. 3:**
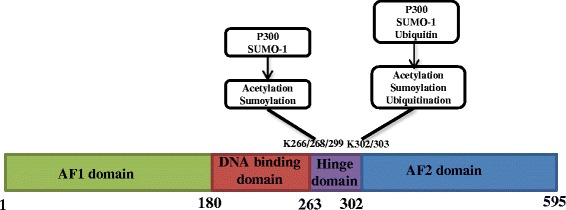
The known ERα protein acetylation, sumoylation and ubiquitination sites and their corresponding enzymes. The Activator Function 1 (AF1) domain at the N-terminal of the ERα protein can transactivate transcription in the absence of ligand binding. The DNA-binding domain (DBD) binds to estrogen response elements (EREs) in DNA. The AF2 domain is the ligand-dependent transactivation domain. As part of its transactivation function, the AF2 domain also binds to several co-activators and co-repressors of ERα

ERα protein activity can be regulated by various post-translational modifications. The known modifications include phosphorylation, ubiquitination, sumoylation, acetylation, methylation and O-linked N-acetylglucosamine. The many sites of modifications are widely distributed over the ERα protein (Fig. 3). Modifications of ERα protein can modulate its functions in several ways. For example, acetylation in the hinge domain of ERα changes the ligand sensitivity and subsequent histone de-acetylation effect [53]. For example, p300 is shown to acetylate ERα protein on the DBD (DNA binding domain), which is shown to enhance ERα activity [53]. Phosphorylation of ERα increases its interaction with ERα co-activators [54]. For example, Tharun et al. showed that phosphorylation at Y537 of ERα changed the helix loop conformation and subsequently increased ligand or co-factor binding efficacy [55]. In addition, many ERα protein modifiers could act as co-activators, which co-occupy with ERα on promoter regions, such as p300 and PIAS [56, 57]. Hanstein et al. first reported that p300 interacts with ERα as an important co-activator [58]. Several years later, Wang et al. reported p300 as an acetylation ligase on ERα and that the acetylation effect enhanced ERα transcriptional activity [53].

**Fig. 4:**
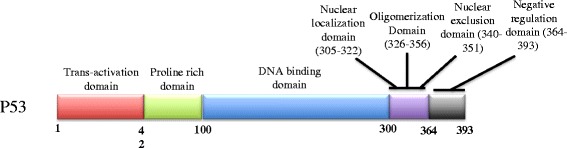
P53 protein domain structure. The N-terminal part amino acids 1–42, constitutes the transactivation domain. The proline-rich domain, from amino acid 42 to amino acid 100, is proven necessary for p53 dependent apoptosis and cell cycle arrest. The DBD (DNA binding domain) is rich in arginine and related to transcriptional activity. The protein domain from amino acid 305 to amino acid 322 includes the nuclear localization domain. The domain from amino acid 340 to amino acid 351 includes the nuclear exclusion domain. In addition, the protein domain from amino acid 326 to amino acid 356 corresponds to the tetramerization domain. The C-terminal domain from amino acid 364 to amino acid 393 is required for DNA binding capability and DNA damage response

Besides ERα, P53 is another star protein in breast cancer area. The p53 protein is encoded by the TP53 gene, which is located on chromosome 17 [59]. Structural and functional analysis reveals that p53 is composed of several functional domains (Fig. 4). The p53 protein is activated by several events, such as DNA damage, oxidative stress and oncogene activation [60, 61]. If activated, the p53 half-life will increase, leading to activation of p53 target genes [62]. Several p53 target genes, including p21, are involved in cell cycle arrest [63]. Another group of target genes regulate cell apoptosis, including the BAX and Fas proteins [64]. In addition to its transactivation function, p53 exerts trans-repression functions on several oncogenes, such as bcl-2 [65]. P53 is also reported to mediate DNA repair via interaction with DNA repair proteins, such as BRCA1 and ATM [66, 67].

P53 is under precise control in unstressed conditions. If the p53 pathway is not activated, the p53 half-life is approximately 20 min, regulated mainly by ubiquitination and proteasomal degradation [68]. Several ubiquitination sites are found at the C-terminal domain of p53, including K370/K372/K373/K381/K382/K386 [69]. Several E3 ubiquitin ligases have been shown to directly poly-ubiquitinate the p53 protein and induce its proteasomal degradation, including MDM2, COP1 and Pirh2 [70]. The most studied of these is the MDM2 protein. MDM2 is a direct target gene of p53 [71]. When p53 is activated, it will induce the expression of MDM2. The MDM2 protein will interact with p53 at the N-terminus and block its transcriptional function [72]. MDM2 also facilitates poly-ubiquitination at several lysine residues in the p53 DBD and C-terminus, which subsequently induces the degradation of p53 [73–75]. This MDM2-p53 negative feedback effectively keeps the cells responding appropriately to certain stimulus [73]. Besides this cross talk between MDM2 and p53, more and more E3 ubiquitin ligases are found to modify the MDM2-p53 complex and indirectly regulate p53 poly-ubiquitination and degradation, including RNF2 and Smurf [76, 77]. E3 ubiquitin ligases that indirectly modify p53 are highly expressed in cancers and thought to be involved in carcinogenesis by suppressing p53 function [78].

RNF31, which was firstly identified from MCF-7 cells, is found to be necessary in estradiol stimulated cell proliferation [20, 40]. Further experiments showed that RNF31 depletion significantly decreased ERα protein level, ERα target gene expression, ERα-regulated reporter gene activity and ERα recruitment to the promoter regions of target genes. Analysis of breast cancer samples reveals that RNF31 is highly expressed in breast tumors compared with adjacent tissues, while RNF31 expression is correlated with ERα target genes both in cell line and in clinical samples. Mechanistic studies showed that RNF31 interacts with ERα and increases its protein stability through RBR domain. Besides, our results demonstrate that RNF31 increased mono-ubiquitination of ERα, and this was dependent on the RBR domain and the E3 ligase activity.

**Fig. 5:**
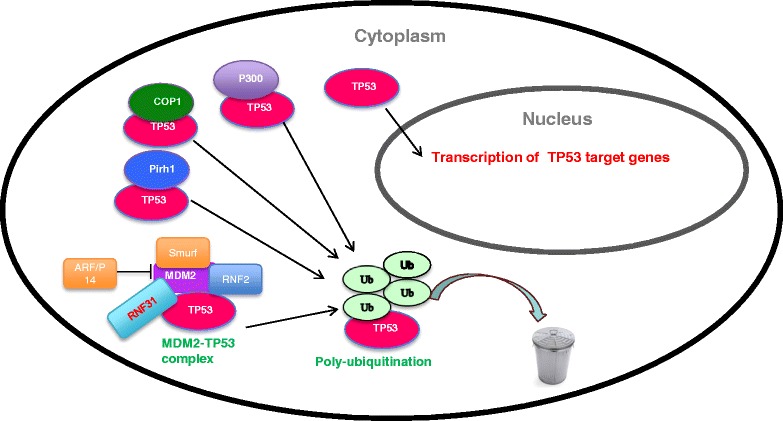
The regulatory effect of RNF31 and other E3 ligases on P53. RNF31 interacts with P53/MDM2 complex and facilitates P53 degradation in MDM2 dependent manner

In the microarray analysis based on MCF-7 cells, we observed that the p53 pathway is significantly affected upon RNF31 knockdown [19]. Our results further show that RNF31 depletion decreased the fraction of proliferating cells in the MCF-7 and ZR-75-1 cell lines. Using dual staining with Annexin V and PI, we found that knockdown of RNF31 facilitated cisplatin-induced apoptosis, while knockdown of p53 in addition to knockdown of RNF31 rescued this effect. This supports that interaction of RNF31 and p53 inhibits apoptosis. Measurement of p53 half-life revealed that RNF31 mainly regulated p53 stability in MDM2 dependent manner. Further experiments showed that RNF31 affected MDM2 stability and proteasomal degradation by inhibiting MDM2 poly-ubiquitination. However, it is not clear how RNF31 affect the poly-ubiquitination of MDM2. There are several possible explanations: RNF31 may compete with other E3 ligases and inhibit MDM2 degradation. Another possibility is that RNF31, as atypical E3 ligase, could function to increase MDM2 stability through mono-ubiquitination. More research is needed to elucidate the regulatory function of RNF31 on MDM2 (Fig. 5).

It is well established that functional p53 is necessary for chemotherapy-induced cell death. One approach, which increases the efficacy of chemotherapy, is to increase p53 protein levels [79]. In our study, we report that RNF31 depletion can arrest the cell cycle and enhance cisplatin-induced cell death. This study uncovers a potential oncogenic role of RNF31: the suppression of p53 signaling. As such, RNF31 could be a potential target to increase the efficacy of chemotherapy. Further, we provided additional knowledge of the molecular mechanism underlying regulation of p53 signaling in breast cancer cells.

### RNF31: the future study and possible therapeutic targets for breast cancer

Since RNF31 was firstly cloned from MCF-7 cells, there is accumulating knowledge about this protein, including protein structure, domain function, pathway regulation and relation with disease [19, 20, 40, 41, 50]. The most striking finding is the observation of RNF31 involving in linear ubiquitination assembly complex in NFκB signaling [46] (Fig. 2). As RNF31 whole knockout leads to embryonic death, it may indicate the important function in development [80]. Since the oncogenic genes always involves in development, RNF31 may be an important oncogenic gene participating carcinogenesis and tumor evolution.

The functional role of RNF31 in human cancer is not thoroughly studied. Currently, RNF31 was found to facilities lymphoma growth through NFκB pathway [50]. Another study also showed that RNF31 mediated cisplatin resistance in multiple cancer cell models [49]. In our previous studies, we found the oncogenic role of RNF31 in breast cancer growth through facilitating ERα signaling and suppressing P53 signaling [19, 20]. Besides, our microarray data also indicated a few novel pathways affected in breast cancer cells, including NFκB pathway, TGFβ pathway and Wnt pathway [20]. As p53 wild type tends to appear in ERα positive breast cancers (Luminal A and B), there is still little known about RNF31 in HER2 type or triple negative breast cancers. Interestingly, our unpublished data already indicates the intriguing phenotype in triple negative breast cancers.

It is exciting to know the development of RNF31 inhibitor is ongoing. A lymphoma study showed that the blocking peptide targeting UBL domain of RNF31 significantly inhibited lymphoma proliferation [81]. However, there are still several limitations for this peptide. One is that since UBL domain is necessary for linear ubiquitination function of RNF31, it might not affect the function of RNF31 on ERα and p53 signaling based on our current results. Another is whether the penetration ability of the peptide is enough to maintain the function in the cytoplasm for breast cancer cells. Besides, compared with small molecular inhibitors, the peptide would be more costly, less stable and possible immune reaction. Since it is already known that the lysine residues on RBR domain is necessary for the E3 ligase function of RNF31, it will be interesting to develop the inhibitors targeting on the E3 ligase function.

## Conclusion

The knowledge of RNF31 and its role in breast cancer is still very limited. We propose that RNF31 mono-ubiquitinatesERα. However, other studies propose that RNF31 can mediate linear ubiquitination in several other models. Since RNF31 is an atypical ubiquitin ligase, its different ubiquitination patterns to differentsubstrates should be thoroughly investigated. Additionally, since we only characterize the role of RNF31 in supporting estrogen signaling and inhibiting P53 signaling in ERα-positive breast cancer cells, further investigation is required to characterize the role of RNF31 in TNBC cells, which are ERα negative and express mutant P53. Moreover, since review focused more on the molecular mechanisms of RNF31, future studies should focus more on the development of drug targets and clinical applications.

## Abbreviations

BMP, bone morphogenetic proteins; DBD, DNA - binding domain; ERα/ESR1, estrogen receptor α; H2AX, H2A Histone Family Member X; HECT, homologous to the E6-AP carboxyl terminus; HER2, human epidermal growth factor receptor 2; IKK_γ_, IκB kinase γ; LUBAC, linear ubiquitin chain assembly complex; NFκB, nuclear factor kappa B; p53/TP53, tumor protein p53; PR, progesterone receptor; RBR domain, RING-IBR-RING domain; RNF, RING finger protein; UBA domain, ubiquitin binding associated domain; ZNF-RBZ, zinc finger domain in Ran-binding proteins and other proteins.
